# Assessment of health‐related quality of life and health utilities in Australian patients with cirrhosis

**DOI:** 10.1002/jgh3.12462

**Published:** 2020-12-10

**Authors:** Steven M McPhail, Samath Amarasena, Katherine A Stuart, Kelly Hayward, Rohit Gupta, David Brain, Gunter Hartel, Tony Rahman, Paul J Clark, Christina M Bernardes, Richard Skoien, Benjamin Mckillen, Andrew Lee, Leshni Pillay, Lei Lin, Myat Myat Khaing, Leigh Horsfall, Elizabeth E Powell, Patricia C Valery

**Affiliations:** ^1^ Australian Centre for Health Services Innovation and Centre for Healthcare Transformation, School of Public Health and Social Work Queensland University of Technology Brisbane Queensland Australia; ^2^ Clinical Informatics Directorate Metro South Health Brisbane Queensland Australia; ^3^ Department of Gastroenterology and Hepatology Royal Brisbane and Women's Hospital Brisbane Queensland Australia; ^4^ Department of Gastroenterology and Hepatology Princess Alexandra Hospital Brisbane Queensland Australia; ^5^ Centre for Liver Disease Research, Translational Research Institute, Faculty of Medicine The University of Queensland Brisbane Queensland Australia; ^6^ Gastroenterology and Hepatology Department The Prince Charles Hospital Brisbane Queensland Australia; ^7^ QIMR Berghofer Medical Research Institute Brisbane Queensland Australia; ^8^ Department of Gastroenterology and Hepatology Mater Hospitals Brisbane Queensland Australia; ^9^ Department of Gastroenterology and Hepatology Logan Hospital Brisbane Queensland Australia

**Keywords:** chronic liver disease, health utilities, quality of life, short form‐36

## Abstract

**Background and Aim:**

Health‐related quality‐of‐life measurements are important to understand lived experiences of patients who have cirrhosis. These measures also inform economic evaluations by modelling quality‐adjusted life years (QALYs). We aimed to describe health‐related quality of life, specifically multiattribute utility (scale anchors of death = 0.00 and full health = 1.00), across various stages and etiologies of cirrhosis.

**Methods:**

Face‐to‐face interviews were used to collect Short Form 36 (SF‐36) questionnaire responses from CirCare study participants with cirrhosis (June 2017 to December 2018). The severity of cirrhosis was assessed using the Child‐Pugh score classified as class A (5–6 points), B (7–9), or C (10–15) and by the absence (“compensated”) *versus* presence (“decompensated”) of cirrhosis‐related complications.

**Results:**

Patients (*n* = 562, average 59.8 years [SD = 11.0], male 69.9%) had a range of primary etiologies (alcohol‐related 35.2%, chronic hepatitis C 25.4%, non‐alcoholic fatty liver disease (NAFLD) 25.1%, chronic hepatitis B 5.9%, “other” 8.4%). Significantly lower (all *P* < 0.001) mean multiattribute utility was observed in the health states of patients with decompensated (mean = 0.62, SD = 0.15) *versus* compensated cirrhosis (mean = 0.68, SD = 0.12), Child‐Pugh class C (mean = 0.59, SD = 0.15) or B (mean = 0.63, SD = 0.15) *versus* A (mean = 0.68, SD = 0.16), and between those of working age (18–64 years; mean = 0.64, SD = 0.16) *versus* those aged 65+ years (mean = 0.70, SD = 0.16). The greatest decrements in health‐related quality of life relative to Australian population norms were observed across physical SF‐36 domains.

**Conclusions:**

Persons with more advanced cirrhosis report greater life impacts. Estimates from this study are suitable for informing economic evaluations, particularly cost‐utility modelling, which captures the benefits of effective prevention, surveillance, and treatments on both the quality and quantity of patients' lives.

## Graphical abstract

Measuring health‐related quality of life is important for understanding patients' experiences living with cirrhosis. Multiattribute utility is a summary measure commonly derived from health‐related quality‐of‐life questionnaires by applying population‐based preference weights with anchors of death (0.00) and perfect health (1.00) and is used in health state modelling, including economic evaluations that incorporate quality‐adjusted life years (known as QALYs). Here, we report health‐related quality‐of‐life and multiattribute utility values for a range of cirrhosis disease stages. These values will inform both clinical and economic research, including cost‐effectiveness modelling that captures benefits to both the quantity and quality of people's lives through the better prevention and management of cirrhosis.

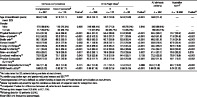

## Introduction

People living with cirrhosis often experience undesirable life impacts, including reduced capacity to undertake daily activities and work, interruptions to sleep patterns, and elevated levels of stress and anxiety.^1^, ^2^, ^3^ Progression of cirrhosis can also be accompanied by a greater decrement in quality of life.^4^

In recent decades, we have seen increased recognition of the importance of patient‐centered care in clinical practice, clinical research, and economic evaluation of new models of care. This includes greater use of patient‐reported outcomes, including use of condition‐specific and generic measures of health‐related quality of life in clinical practice and research. Use of condition‐specific patient‐reported outcomes has the potential to inform our understanding of patients' health profiles as they relate to the need for medical interventions and supportive care relevant to patients' conditions,^5^ as well as potential changes in patients' lived experiences of chronic conditions over time in response to treatments or disease progression.^6^, ^7^ Generic measures of health‐related quality of life are also useful in clinical practice and research contexts for understanding key areas of health‐related quality of life that may be impacted by a patient's liver disease, other health conditions, or as a result of the combined impact of multimorbidity in patients with complex chronic disease.^7^, ^8^ In response, clinical teams, in partnership with patients, may use these patient‐reported outcomes as a foundation for the design and implementation of patient‐centered approaches to clinical and supportive care provision.^9^

The principle of evaluating the effects on quality of life from patients' own perspective has also become a primary consideration in economic evaluations. In cost‐utility analysis, which is considered a special case of cost‐effectiveness analysis, the “effect” is quantified through the use of quality‐adjusted life years (QALYs) that can be indirectly derived from widely used generic health‐related quality‐of‐life questionnaires. In this context, an algorithm that has been derived from the health state preferences of the population is applied to questionnaire responses to produce a “multi‐attribute utility” index, sometimes referred to as a “utility score,” “utility index,” “health utility,” or simply “utility,” which is the term we will use throughout this manuscript. Utility is expressed on a scale where death is represented by 0.00 and perfect health is represented by 1.00. A QALY is the equivalent of accruing 1 year with a full utility score, for example, living 2 years in a health state assigned a utility value of 0.50. This enables economic evaluations comparing two or more interventions to take into account not only the effect on quantity of life but also the quality of the lived experience from the perspective of the person completing the generic health‐related quality‐of‐life questionnaire.^10^

Economic evaluations are important for informing resource allocation decision‐making in health system environments where demand for health services is increasing at faster rates than available health‐care resources.^11^ Evidence from an economic perspective is increasingly well regarded by health‐care decision‐makers, particularly by those who wish to maximize health gain per dollar invested.^12^ To ensure that hepatology and gastroenterology services are resourced appropriately, particularly for progressive conditions including cirrhosis, it is important to report findings related to the cost‐effectiveness of specific interventions or models of care that take into account benefits to patients' quality of life.

There are currently barriers to conducting economic modelling for patients with, or at risk of, cirrhosis where modelling of cirrhosis‐related disease states are required. This is particularly true of cost‐utility analyses where effects are derived from quality‐of‐life estimates. There is a paucity of literature related to this topic among people living with cirrhosis. A number of studies can be found regarding the assessment of health‐related quality of life of people living with liver disease,^13^, ^14^, ^15^, ^16^ but they do not specifically quantify health utilities using methods that are useful in economic modelling. Estimates of utility values for a range of relevant health states for people living with cirrhosis (including across stages of disease progression) derived from state‐of‐the‐art economic modelling studies of sufficiently large samples with indicators of central tendencies and distribution are particularly useful but are not widely available at present. To address this gap, we aimed to report health‐related quality of life and associated utility values for stages of disease severity among people living with cirrhosis from a large cohort study for future use in both clinical and economic research. In addition to advancing our understanding of the health profile of people living with cirrhosis from their own perspective, the utility estimates provide valuable evidence for analysts conducting economic evaluations to ensure appropriate allocation of resources to services for people with liver disease, including clinical interventions that prevent or slow the progression of cirrhosis.

## Methods

### *Design, setting and participants*

The CirCare study is a multicenter cohort study of patients with cirrhosis recruited from five hospitals in Brisbane, Queensland from June 2017 to December 2018.^5^ Consecutive adult patients identified from selected ambulatory Hepatology/Gastroenterology clinic appointment lists or admitted with a diagnosis of cirrhosis were eligible to participate. Patients were excluded if their treating clinician considered them to have a cognitive or physical impairment that could interfere with participation or if they were unable to communicate in English and an interpreter was not available to assist with the interview. A study nurse and a hepatologist assessed patients' eligibility, and the study nurse obtained written consent for participation in the study.

### *Data collection and measurements*

Patient characteristics and self‐reported assessments were collected using structured questionnaires via “face‐to‐face” interviews at recruitment, with the exception of a small number (*n* = 47; 8.4%) of patients who, due to pragmatic constraints in the clinical setting (e.g. patient had to return to work, patient scheduled for imaging or a procedure), had part of their data collected using self‐administered questionnaires. Clinical data were obtained from the patients' medical records.

Severity of liver disease was measured by calculating the Child‐Pugh score on the day of recruitment (classified as class A [5–6 points], B [7–9], or C [10–15]), and by the absence (“compensated”) *versus* presence (“decompensated”) of cirrhosis‐related complications at recruitment, namely, ascites, hepatic encephalopathy, variceal bleeding, and jaundice. Comorbidity burden at the time of recruitment was measured using the Charlson Comorbidity Index (CCI).^17^ Using validated coding algorithms,^18^ binary indicators were created for each condition (present/absent), which were used to calculate the CCI score (a weighted sum based on the presence and severity of comorbidities present).^17^ CCI score was categorized as score “0” (no known comorbidity), “1–2,” and “3+,” with higher scores indicating higher comorbidity burden.

### *Health‐related quality of life and health utilities*

The Short Form 36 (SF‐36)^19^ questionnaire containing 36 questions grouped into eight domains was used to assess the health‐related quality‐of‐life profile of participants. SF‐36 domains comprise general health, physical functioning, social functioning, pain, role limitations due to physical problems (role‐physical), emotional well‐being, role limitations due to emotional problems (role‐emotional), and vitality. Raw domain scores were transformed to range from 0 to 100, with a higher score indicating a higher quality of life. The domain scores were normalized to the Australian population means and SD^20^ to calculate the Physical Component Summary (PCS) and Mental Component Summary (MCS) scores using weights reported from factor analysis of the SF‐36 items and with adjustment for interitem correlations.^21^ PCS and MCS scores were transformed to have a mean of 50 and an SD of 10. For each patient, the health utility score was also calculated from relevant SF‐36 items that comprise the Short Form 6D (SF‐6D).^22^

### *Data analyses*

Analyses were conducted using Stata/SE (Version 15; Stata Corporation, College Station, TX, USA). Descriptive analyses are presented as frequency (percentages, %) and mean (SD). Differences between groups were assessed using the Chi‐squared test for categorical variables and linear regression (adjusted for age) for continuous variables. The independent‐samples t‐test was used to compare SF‐36 domains^20^ and utility values^23^ against Australian population norms that were age‐ and gender‐adjusted to match the study sample. Correlations between the Child‐Pugh score and subscales of the SF‐36^19^ were examined using Spearman's rank correlation coefficients (Spearman's rho are reported). Statistical significance was set at alpha = 0.05, and all *P*‐values were two‐sided.

## Results

**Figure 1:**
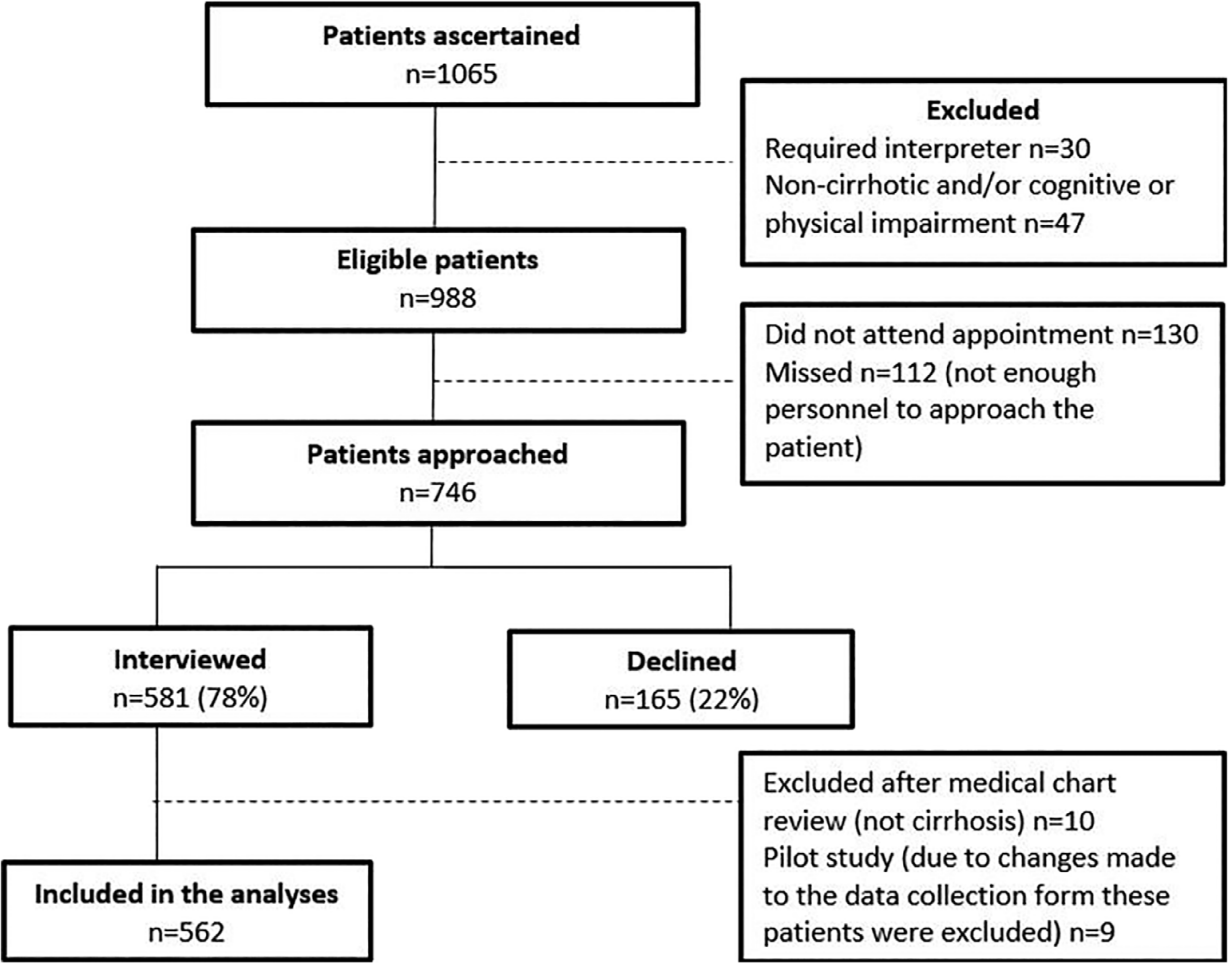
Recruitment process.

A total of 1065 patients with cirrhosis were identified during the recruitment period, with 746 invited to participate in the study (581 interviewed; 165 declined). Patients who failed to attend clinic (*n* = 130), who attended when there was not enough personnel to approach the patient (“missed,” *n* = 112), and who were ineligible (*n* = 77) were not invited to participate (Fig. 1). The study data collection form was piloted among nine patients. Due to changes made to the data collection form, these nine patients were excluded from the analysis. An additional 10 patients were excluded after medical chart review determined they did not have cirrhosis. Data from 562 patients included in the final analysis are described hereafter.

Most patients were recruited via ambulatory Hepatology/Gastroenterology clinics (*n* = 479, 85.2%), and 83 patients (14.8%) were admitted to hospital at the time of recruitment. The latter were interviewed while in hospital; they had no physical impairment that could interfere with participation. Just over half the patients were recruited from the Princess Alexandra Hospital (*n* = 315, 56.0%), 18.1% were recruited from the Royal Brisbane and Women's Hospital (*n* = 102), 9.8% from the Prince Charles Hospital (*n* = 55), 8.5% from the Mater Hospital (*n* = 48), and 7.5% from the Logan Hospital (*n* = 42).

The majority of patients were male (*n* = 393, 69.9%) with a mean age of 59.8 years (SD = 11.0) at recruitment; 367 (65.3%) were considered to be of working age (18–64 years). Twenty‐six (4.6%) patients identified themselves as Aboriginal or Torres Strait Islander (Indigenous) Australians. Alcohol‐related cirrhosis was the most common primary etiology (*n* = 198, 35.2%), followed by chronic hepatitis C (*n* = 143, 25.4%), non‐alcoholic fatty liver disease/non‐alcoholic steatohepatitis (NAFLD/NASH; *n* = 141, 25.1%), chronic hepatitis B (*n* = 33, 5.9%), and 47 (8.4%) had other causes (autoimmune liver disease [*n* = 23], metabolic liver disease [*n* = 6], cryptogenic [*n* = 5], biliary atresia [*n* = 3], progressive familial intrahepatic cholestasis [*n* = 3], Budd Chiari syndrome [*n* = 2], Alagille syndrome [*n* = 1], Bardet‐Biedl syndrome [*n* = 1], drug‐induced liver injury [*n* = 1], and etiology uncertain [*n* = 2]).

Most patients had Child‐Pugh class A cirrhosis (*n* = 358, 63.7%) at recruitment; 21.7% (*n* = 122) had class B, and 10.7% (*n* = 60) class C. Child‐Pugh score was not calculated for 22 patients (3.9%) taking warfarin at recruitment. Approximately one‐third (*n* = 175, 31.1%) of patients had at least one cirrhosis‐related complication at recruitment (“decompensated”). At the time of recruitment, 136 patients had ascites (24.2%), 97 (17.3%) were jaundiced, 46 (8.2%) had hepatic encephalopathy, and 10 (1.8%) had variceal bleeding. Approximately one‐third of patients (*n* = 190, 33.8%) had no comorbidity at recruitment; 280 (49.8%) had a CCI of 1–2, and 92 (16.4%) had a CCI of 3+, reflecting a greater number and severity of comorbidities. The most common comorbidity was diabetes, with 42.0% (*n* = 236) of patients having a diagnosis of diabetes documented in their medical notes around the time of recruitment (2 of 236 patients had type 1 diabetes), followed by hypertension (*n* = 207, 36.8%), dyslipidemia (*n* = 136, 24.2%), anxiety or depression (*n* = 134, 23.8%), heart disease (*n* = 83, 14.7%; Charlson Comorbidity Index disease categories ischemic heart disease and congestive heart failure), and lung disease (*n* = 76, 13.5%; Charlson Comorbidity Index disease category chronic pulmonary disease).

**Figure 2:**
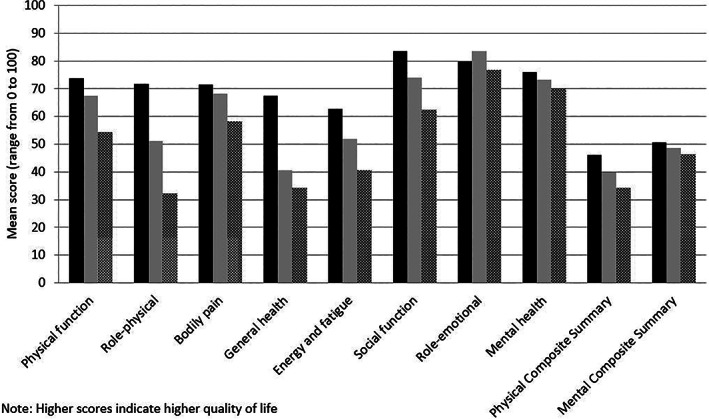
SF‐36 domain mean scores of patients with cirrhosis (compensated and decompensated) *versus* Australian population norms (age and gender adjusted). *Note*: Higher scores indicate higher quality of life. (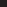), Australian norm; (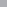), compensated; (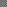), decompensated.

Health‐related quality of life (SF‐36) and utility estimates for patients with cirrhosis at different disease stages compared with Australian population norms are presented in Table 1. Statistically significant differences in most SF‐36 domains and utility values were associated with the presence of cirrhosis‐related complications and Child‐Pugh class. Statistically significant differences in utility scores were also seen according to gender, age groups (18–64 years *vs* 65+ years), Charlson Comorbidity Index, primary disease etiology (three main three groups were compared), and the presence of diabetes (Tables 2, 3, 4, 5 for comparisons). No difference in utility values or SF‐36 domains were seen according to recruitment hospital (data not shown). Statistically significant differences were seen for utility values and for most SF‐36 domains when comparing all patients with cirrhosis against Australian norms. Figure 2 illustrates comparisons between study participants' SF‐36 values and normative Australian values.

**Table 1:** Health‐related quality of life (SF‐36) and health utilities for patients with cirrhosis at different disease stages compared with Australian population norms

	Cirrhosis complications			Child‐Pugh Class^†^				All cirrhosis patients	Australian norm^‡^	
	Compensated	Decompensated^§^		A	B	C				
	*n* = 387	*n* = 175	*P*‐value^¶^	*n* = 358	*n* = 122	*n* = 60	*P*‐value^¶^	*n* = 562	*n* = 18 468	*P*‐value^††^
Age at recruitment (years) mean (SD)	60.8 (10.8)	57.6 (11.1)	0.002	60.5 (10.7)	59.2 (10.9)	54.9 (10.9)	<0.001	59.8 (11.0)	—	—
Gender										
Male	270 (69.8%)	123 (70.3%)	0.900	245 (68.4%)	87 (71.3)	45 (75.0%)	0.540	393 (69.9%)	—	—
Female	117 (30.2%)	52 (29.7%)		113 (31.6%)	35 (28.7%)	15 (25.0%)		169 (30.1%)	—	—
Physical functioning^‡‡^	67.4 (27.8)	54.5 (29.9)	<0.001	68.4 (27.3)	54.4 (29.8)	51.8 (30.9)	<0.001	63.4 (29.1)	73.7 (23.2)	<0.001
Role—physical^‡‡^	51.2 (42.3)	32.3 (37.0)	<0.001	52.3 (42.2)	33.0 (37.7)	27.1 (33.9)	<0.001	45.3 (41.6)	71.8 (34.8)	<0.001
Bodily pain^‡‡^	68.1 (30.6)	58.2 (33.0)	0.001	68.0 (30.0)	60.7 (33.6)	52.6 (33.4)	<0.001	65.0 (31.6)	71.6 (24.8)	<0.001
General health^‡‡^	40.5 (19.9)	34.4 (17.6)	0.003	41.1 (19.6)	34.6 (18.1)	31.2 (16.9)	<0.001	38.6 (19.4)	67.5 (20.4)	<0.001
Energy and fatigue^‡‡^	52.0 (26.4)	40.6 (25.9)	<0.001	52.5 (26.5)	40.9 (25.2)	37.3 (24.4)	<0.001	48.4 (26.7)	62.6 (19.8)	<0.001
Social functioning^‡‡^	74.1 (30.0)	62.4 (35.2)	<0.001	75.0 (30.0)	61.6 (33.3)	58.4 (36.6)	<0.001	70.4 (32.1)	83.6 (22.3)	<0.001
Role—emotional^‡‡^	83.5 (26.1)	76.8 (28.8)	0.017	83.2 (26.1)	79.5 (28.6)	72.3 (28.5)	0.027	81.4 (27.1)	79.9 (32.2)	0.279
Emotional well‐being^‡‡^	73.3 (22.6)	70.1 (23.8)	0.343	73.3 (22.6)	71.8 (21.3)	64.4 (28.1)	0.103	72.3 (23.0)	75.9 (16.9)	<0.001
Physical Composite Summary^‡‡^	39.9 (12.0)	34.4 (10.8)	<0.001	40.3 (11.7)	34.3 (11.4)	33.4 (10.5)	<0.001	38.2 (11.9)	46.1 (10.2)	<0.001
Mental Composite Summary^‡‡^	48.7 (11.8)	46.4 (11.9)	0.174	48.7 (11.8)	47.1 (10.7)	44.0 (13.7)	0.096	48.0 (11.9)	50.7 (10.0)	<0.001
SF6D health utility^§§^	0.68 (0.12)	0.62 (0.17)	<0.001	0.68 (0.16)	0.63 (0.15)	0.59 (0.15)	<0.001	0.66 (0.16)	0.74 (0.12)	<0.001

^†^ Not calculated for 22 patients taking warfarin at recruitment.

^‡^ Australian population age‐ and gender‐adjusted means and SD.^20^, ^23^

^§^ Decompensated cirrhosis defined as patient having at least one cirrhosis‐related complication at recruitment.

^¶^ Linear regression adjusted for age for continuous data and chi‐squared test for categorical data.

^††^ Unadjusted *P*‐value for difference between all patients and Australian norms.

^‡‡^ Missing data ranged from 3 (0.5%) to 6 (1.1%).

^§§^ Missing data for 13 patients (2.3%).

Mean (SD) and frequency (percentage).

**Table 2:** Health‐related quality of life and health utilities for patients with cirrhosis by gender, age group, and first language

	Gender			Age group			First language		
	Female	Male		18–64 years	65+ years		English	Not English	
	*n* = 169	*n* = 393	*P*‐value^†^	*n* = 367	*n* = 195	*P*‐value^†^	*n* = 483	*n* = 79	*P*‐value^†^
Age at recruitment (years) mean (SD)	59.8 (11.2)	59.8 (10.9)	0.971	53.8 (8.3)	71.1 (4.8)	—	59.9 (10.7)	59.3 (12.9)	0.668
Gender									
Male	—	—	—	106 (28.9%)	63 (32.3%)	0.400	144 (29.8%)	25 (31.6%)	0.740
Female				261 (71.1%)	132 (67.7%)		339 (70.2%)	54 (68.4%)	
Physical functioning^‡^	56.0 (30.0)	66.5 (28.1)	<0.001	64.9 (28.5)	60.4 (30.0)	0.078	62.8 (29.2)	66.9 (28.7)	0.268
Role—physical^‡^	44.5 (41.2)	45.7 (41.8)	0.750	41.9 (41.2)	51.8 (41.6)	0.007	44.4 (41.6)	51.0 (41.8)	0.192
Bodily pain^‡^	59.3 (32.4)	67.5 (31.0)	0.005	61.8 (31.9)	71.2 (30.2)	0.001	63.9 (31.8)	71.8 (30.2)	0.042
General health^‡^	37.3 (18.0)	39.2 (20.0)	0.273	35.8 (19.2)	44.0 (18.8)	<0.001	38.0 (19.0)	42.9 (21.6)	0.031
Energy and fatigue^‡^	44.9 (27.1)	50.0 (26.5)	0.037	45.3 (26.9)	54.3 (25.4)	<0.001	46.9 (26.3)	58.0 (27.6)	0.001
Social functioning^‡^	69.0 (32.2)	71.1 (32.2)	0.466	66.8 (32.6)	77.3 (30.3)	<0.001	69.1 (32.5)	78.6 (28.5)	0.013
Role—emotional^‡^	77.4 (28.6)	83.1 (26.3)	0.021	79.6 (27.7)	84.7 (25.6)	0.034	81.5 (26.9)	80.8 (28.2)	0.855
Emotional well‐being^‡^	69.9 (22.6)	73.3 (23.1)	0.098	68.7 (24.0)	79.0 (19.4)	<0.001	71.6 (23.0)	76.2 (23.2)	0.085
Physical Composite Summary^‡^	36.3 (11.9)	39.0 (11.8)	0.012	38.1 (12.0)	38.4 (11.6)	0.784	37.9 (11.9)	40.3 (11.7)	0.105
Mental Composite Summary^‡^	47.2 (11.9)	48.3 (11.9)	0.309	45.9 (12.3)	51.8 (9.9)	<0.001	47.6 (11.7)	50.5 (12.4)	0.029
SF6D health utility^§^	0.64 (0.16)	0.67 (0.16)	0.035	0.64 (0.16)	0.70 (0.16)	<0.001	0.71 (0.12)	0.74 (0.12)	0.006

^†^ Linear regression adjusted for age for continuous data and chi‐squared test for categorical data.

^‡^ Missing data ranged from 3 (0.5%) to 6 (1.1%).

^§^ Missing data for 13 patients (2.3%).

Mean (SD) and frequency (percentage).

**Table 3:** Health‐related quality of life and health utilities for patients with cirrhosis by Charlson Comorbidity Index and diabetes status

	Charlson Comorbidity Index				Diabetes		
	CCI = 0	CCI = 1–2	CCI = 3+		Absent	Present	
	*n* = 190	*n* = 280	*n* = 92	*P*‐value ^†^	*n* = 326	*n* = 236	*P*‐value ^†^
Age at recruitment (years) mean (SD)	55.7 (11.4)	60.5 (9.9)	65.9 (10.2)	<0.001	57.7 (11.4)	62.6 (9.8)	<0.001
Gender							
Male	50 (26.3%)	88 (31.4%)	31 (33.7%)	0.350	90 (27.6%)	79 (33.5%)	0.130
Female	140 (73.7%)	192 (68.6%)	61 (66.3%)		236 (72.4%)	157 (66.5%)	
Physical functioning^‡^	71.4 (27.2)	60.4 (29.5)	56.3 (28.5)	<0.001	66.8 (28.6)	58.6 (29.2)	0.008
Role—physical^‡^	51.7 (42.6)	42.4 (40.5)	41.8 (42.1)	0.006	48.5 (42.3)	41.0 (40.4)	0.015
Bodily pain^‡^	69.6 (30.1)	61.1 (32.6)	68.1 (30.4)	0.005	68.3 (30.5)	60.5 (32.7)	0.001
General health^‡^	41.8 (19.4)	37.9 (19.3)	34.6 (19.1)	<0.001	40.3 (19.8)	36.4 (18.6)	0.001
Energy and fatigue^‡^	51.8 (27.6)	46.3 (26.9)	48.5 (24.4)	0.022	50.4 (27.2)	45.8 (25.9)	0.009
Social functioning^‡^	71.7 (32.1)	70.0 (32.5)	69.6 (31.6)	0.218	72.0 (31.0)	68.4 (33.6)	0.037
Role—emotional^‡^	81.7 (26.4)	81.4 (27.3)	81.5 (27.7)	0.349	81.4 (26.8)	81.4 (27.5)	0.560
Emotional well‐being^‡^	72.3 (23.0)	71.9 (23.4)	73.7 (22.2)	0.270	72.6 (23.2)	71.8 (22.9)	0.147
Physical Composite Summary^‡^	41.5 (11.5)	36.6 (12.1)	36.3 (10.9)	<0.001	39.8 (11.6)	35.9 (11.9)	<0.001
Mental Composite Summary^‡^	47.3 (11.9)	48.1 (12.0)	48.9 (11.5)	0.449	47.9 (11.8)	48.1 (11.9)	0.273
SF6D health utility^§^	0.69 (0.16)	0.64 (0.16)	0.67 (0.16)	0.011	0.68 (0.16)	0.64 (0.16)	0.001

^†^ Linear regression adjusted for age for continuous data and chi‐squared test for categorical data.

^‡^ Missing data ranged from 3 (0.5%) to 6 (1.1%).

^§^ Missing data for 13 patients (2.3%).

Mean (SD) and frequency (percentage).

CCI, Charlson Comorbidity Index.

**Table 4:** Health‐related quality of life and health utilities for patients with cirrhosis by the three main primary etiology groups

	Alcohol	HCV	NAFLD/NASH	
	*n* = 198	*n* = 143	*n* = 141	*P*‐value^†^
Age at recruitment (years) mean (SD)	58.8 (11.5)	57.8 (7.4)	64.8 (9.4)	<0.001
Gender				
Male	51 (25.8%)	22 (15.4%)	62 (44.0%)	<0.001
Female	147 (74.2%)	121 (84.6%)	79 (56.0%)	
Physical functioning^‡^	61.8 (28.7)	73.4 (25.3)	54.8 (29.3)	<0.001
Role—physical^‡^	44.6 (41.0)	48.4 (43.2)	43.9 (40.6)	0.428
Bodily pain^‡^	65.8 (30.3)	67.2 (33.6)	58.8 (32.1)	0.010
General health^‡^	38.8 (18.2)	42.3 (20.4)	35.6 (18.9)	0.001
Energy and fatigue^‡^	47.7 (26.5)	51.7 (26.8)	44.5 (24.1)	0.011
Social functioning^‡^	70.5 (33.9)	70.8 (31.5)	68.5 (32.4)	0.251
Role—emotional^‡^	80.8 (27.4)	80.3 (27.0)	82.6 (27.0)	0.998
Emotional well‐being^‡^	71.3 (22.9)	71.7 (24.9)	72.2 (21.7)	0.523
Physical Composite Summary^‡^	38.2 (11.4)	41.3 (12.0)	35.0 (12.0)	<0.001
Mental Composite Summary^‡^	47.7 (12.3)	47.0 (12.6)	48.5 (11.1)	0.777
SF6D health utility^§^	0.66 (0.15)	0.68 (0.17)	0.63 (0.16)	0.002

^†^ Linear regression adjusted for age for continuous data and chi‐squared test for categorical data.

^‡^ Missing data ranged from 3 (0.5%) to 6 (1.1%).

^§^ Missing data for 13 patients (2.3%).

Mean (SD) and frequency (percentage).

HCV, hepatitis C virus; NAFLD, non‐alcoholic fatty liver disease; NASH, non‐alcoholic steatohepatitis.

**Table 5:** Health‐related quality of life and health utilities for patients according to specific complications of cirrhosis

	Ascites			Hepatic encephalopathy			Variceal bleeding			Jaundice		
	Absent	Present		Absent	Present		Absent	Present		Absent	Present	
	*n* = 426	*n* = 136	*P*‐value^†^	*n* = 516	*n* = 46	*P*‐value^†^	*n* = 552	*n* = 10	*P*‐value^†^	*n* = 465	*n* = 97	*P*‐value^†^
Age at recruitment (years) mean (SD)	60.0 (11.3)	59.2 (9.9)	0.459	60.0 (11.0)	57.3 (10.2)	0.107	59.7 (11.0)	61.5 (10.0)	0.626	61.0 (10.5)	53.9 (11.3)	<0.001
Gender												
Male	127 (29.8%)	42 (30.9%)	0.810	159 (30.8%)	10 (21.7%)	0.200	166 (30.1%)	3 (30.0%)	1.000	140 (30.1%)	29 (29.9%)	0.970
Female	299 (70.2%)	94 (69.1%)		357 (69.2%)	36 (78.3%)		386 (69.9%)	7 (70.0%)		325 (69.9%)	68 (70.1%)	
Physical functioning^‡^	67.3 (28.0)	51.2 (29.3)	<0.001	64.6 (28.5)	49.5 (32.2)	<0.001	63.8 (28.8)	39.0 (37.0)	0.008	64.6 (28.6)	57.4 (31.0)	0.002
Role—physical^‡^	50.2 (42.2)	30.2 (35.9)	<0.001	47.0 (42.0)	27.2 (32.0)	0.002	45.8 (41.5)	22.5 (41.6)	0.076	48.1 (42.1)	32.3 (36.6)	0.001
Bodily pain^‡^	68.1 (30.2)	55.2 (34.0)	<0.001	66.6 (30.9)	47.3 (34.7)	<0.001	65.4 (31.5)	41.8 (31.0)	0.017	66.0 (31.4)	60.1 (32.4)	0.163
General health^‡^	40.2 (19.7)	33.6 (17.7)	0.001	39.3 (19.6)	30.7 (15.9)	0.009	38.6 (19.3)	38.0 (26.7)	0.851	39.8 (19.6)	33.0 (17.7)	0.027
Energy and fatigue^‡^	51.5 (26.3)	38.6 (26.0)	<0.001	50.0 (26.5)	31.2 (24.0)	<0.001	48.7 (26.6)	34.0 (33.9)	0.075	49.8 (26.8)	41.7 (25.5)	0.030
Social functioning^‡^	73.6 (30.2)	60.6 (36.1)	<0.001	71.9 (31.4)	53.6 (36.3)	0.001	70.9 (31.7)	46.3 (47.5)	0.013	71.9 (31.5)	63.4 (34.6)	0.112
Role—emotional^‡^	83.4 (26.0)	75.1 (29.6)	0.002	82.3 (26.7)	71.0 (29.5)	0.010	81.7 (27.0)	66.7 (31.4)	0.073	82.4 (26.7)	76.4 (28.6)	0.151
Emotional well‐being^‡^	73.4 (22.3)	68.7 (25.0)	0.051	73.0 (22.6)	63.8 (26.0)	0.022	72.5 (23.0)	62.0 (21.4)	0.124	72.7 (22.8)	70.1 (24.1)	0.940
Physical Composite Summary^‡^	39.7 (11.9)	33.3 (10.3)	<0.001	38.7 (11.8)	32.2 (11.0)	<0.001	38.3 (11.8)	29.8 (12.0)	0.026	38.8 (12.0)	35.2 (10.9)	0.001
Mental Composite Summary^‡^	48.6 (11.6)	45.9 (12.4)	0.028	48.4 (11.7)	42.9 (12.7)	0.008	48.1 (11.8)	43.1 (14.1)	0.146	48.3 (11.8)	46.2 (11.9)	0.808
SF6D health utility^§^	0.68 (0.16)	0.61 (0.15)	<0.001	0.67 (0.16)	0.57 (0.14)	<0.001	0.66 (0.16)	0.54 (0.17)	0.016	0.67 (0.16)	0.63 (0.15)	0.045

^†^ Linear regression adjusted for age for continuous data and chi‐squared test for categorical data.

^‡^ Missing data ranged from 3 (0.5%) to 6 (1.1%).

^§^ Missing data for 13 patients (2.3%).

Mean (SD) and frequency (percentage).

There was a significant, although weak‐to‐fair, correlation between Child‐Pugh score (as a continuous score) and five subscales of the SF‐36, namely, physical functioning (Spearman rho = −0.31, *P* < 0.001), role‐physical (Spearman rho = −0.29, *P* < 0.001), energy and fatigue (Spearman rho = −0.27, *P* < 0.001), general health (Spearman rho = −0.24, *P* < 0.001), and social functioning (Spearman rho = −0.24, *P* < 0.001). Correlation was poor (<0.20) for the subscales of bodily pain, role‐emotional, and mental health subscales.

## Discussion

This study describes health‐related quality‐of‐life profiles and provides utility values for people living with the effects of cirrhosis across a range of health states and cirrhosis severity. As expected, more severe disease was associated with lower quality of life, particularly in physical domains, which this study has quantified. Cirrhosis‐related complications and comorbidity burden (particularly comorbid diabetes) were associated with lower utility value point estimates. It was interesting to note that living with cirrhosis at a younger age was associated with a relatively greater decrement in quality of life than living with cirrhosis at older ages. This may be attributable to different lifestyle requirements or expectations of patients at younger ages or the impact of liver disease in the context of work or family commitments, compared with patients living with cirrhosis at more advanced ages. The estimates reported in this study, including those in Supplementary Tables, are likely to be useful for those seeking to model the benefits associated with more effective prevention, surveillance, and treatment when conducting cost‐utility modelling.

Findings from this study generally support previous literature in the field. Several prior studies across a variety of geographic regions have indicated that patients living with cirrhosis have poorer quality of life.^4^, ^24^ The present study extends our current knowledge by reporting utility estimates among key disaggregated health states, including for patients with and without decompensated cirrhosis, across Child‐Pugh classification categories, in the presence or absence of comorbidity, in males *versus* females, and across age categories. It was interesting to note that there was no significant association between Child‐Pugh scores and health‐related quality‐of‐life domain scores for bodily pain, role‐emotional, and mental health.

Utility estimates for people living with decompensated cirrhosis were low (mean 0.62) but considerably higher than those estimated by physicians in a previous study of people with decompensated cirrhosis (mean 0.55).^25^ There are several possible explanations for this finding. First, physicians may genuinely underestimate the health‐related quality of life of people living with decompensated cirrhosis. This explanation has some support in prior studies of proxy‐reporting across a range of clinical populations which indicate that health professionals may document lower values than their patients' self‐report.^26^, ^27^ Second, it is possible that methodological differences used to elicit utility estimates (time‐tradeoff *vs* questionnaire with weights applied in the present study) may, in part, explain these differences.^25^ Third, patients living with cirrhosis may have developed an adaptive response due to their experiences living with a chronic condition that could alter the way they conceptualize and priorities components of their quality of life or subconsciously recalibrate the scale on which they report their health‐related quality of life. This is sometimes collectively referred to as the “response shift phenomenon.”^6^, ^28^, ^29^ Regardless of the relative contribution of these potential explanatory processes, this discrepancy highlights the importance of seeking responses directly from patients when clinical teams or researchers are seeking to understand patients' perspective of their health‐related quality of life.

This study has important implications for modelling health‐related quality of life of future health states among people living with, or at risk of, chronic liver conditions associated with cirrhosis when conducting cost‐effectiveness modelling studies. The reporting of point estimates (and SD) of health‐related quality‐of‐life profiles across SF‐36 domains and utility scores for a range of disaggregated health states will enable statistical modelling of these health states that more accurately represent the perceptions of patients living in these various disease stages, including the presence or absence of comorbidity. This will likely lead to more accurate cost‐effectiveness (or specifically cost‐utility) modelling that takes into account the potential benefit of preventing or delaying transitions to more severe disease stages.

This study included a relatively large sample of patients with a reliable source of clinical data, including assessment of disease severity and etiology by hepatologists, and with a range of disease states consistent with population‐based data on cirrhosis from the underlying population from which this sample was drawn.^30^ The proportion of males and females, age distribution, and etiology were consistent with prior research reported in the liver disease literature.^1^, ^4^, ^5^, ^15^, ^16^, ^30^ This distribution is an important consideration for those seeking to model quality of life among people living with liver disease as males tend to report slightly higher utility than females. Another advantage of the sampling approach was that it included a range of disease etiologies that are likely to be representative of the clinical population presently accessing hepatology services.

Limitations of this study include that it was conducted in a metropolitan area of a high‐income country where access to specialist hepatology services is readily available and where social welfare exists for those who are unable to work due to illness or disability. An important methodological consideration was that utility estimates in the present study were derived from SF‐6D items, a subset of questions from the SF‐36 instrument. This was a pragmatic approach as the SF‐36 instrument was completed to also provide a broader health‐related quality‐of‐life profile. It is noteworthy that other instruments may be used to derive utility estimates in this setting, including the EQ‐5D^31^ and AQOL,^32^ and that utility estimates from alternative instruments may not be entirely consistent with each other.^33^, ^34^ Inconsistency in utility estimates from different instruments may be due in part to the use of different utility elicitation methods when population preference weights were originally developed for each instrument and population. Although there is no singularly preferred method for utility elicitation, the standard gamble approach was used to derive the population preference weights applied to SF‐6D.^22^ It is plausible that this may contribute to inflation of utility values assigned to very poor health states due to risk aversion that has been associated with the standard gamble approach to utility elicitation underpinning SF‐6D utility values.^23^, ^35^ Findings may therefore not be generalizable to dissimilar instruments or settings.

Priorities for future research include the addition of cost‐utility analyses alongside studies evaluating the impact of new prevention, surveillance, and treatment regimens or models of care for delivering services to people with liver disease. To this end, modelling studies that seek to understand both current and future benefits associated with new approaches to prevention, surveillance, and treatment are now able to draw on utility and health‐related quality‐of‐life profiles reported in this study.

## Supporting information


**Appendix** **S1**. Supporting information.
