# Supplementary material for: Assessment of health‐related quality of life and health utilities in Australian patients with cirrhosis
Source: JGH Open. 2020 Dec 10;5(1):133–42. doi: 10.1002/jgh3.12462 (PMC7812472; doi:10.1002/jgh3.12462)
Supplement: Supplementary file 1 — Appendix S1. Supporting information. [file JGH3-5-133-s001.docx]

SUPPLEMENTARY TABLES WERE INCORPORATED IN THE MAIN TEXT
